# Treatment Selection and Prioritization for the EJS ACT‐PD MAMS Trial Platform

**DOI:** 10.1002/mds.30190

**Published:** 2025-04-18

**Authors:** Cristina Gonzalez‐Robles, Dilan Athauda, Thomas R. Barber, Roger A. Barker, David T. Dexter, Susan Duty, Romy Ellis‐Doyle, Sonia Gandhi, Joel Handley, Edwin Jabbari, Keith Martin, Kevin McFarthing, Georgia Mills, Heather Mortiboys, Stephen Mullin, Rebecca Petty, Esther Sammler, Paula Scurfield, Simon R.W. Stott, George K. Tofaris, Li Wei, Caroline H. Williams‐Gray, Alan Wong, Marie‐Louise Zeissler, Richard K. Wyse, Camille B. Carroll, Thomas Foltynie, Oliver Bandmann, Anthony H.V. Schapira, the EJS ACT‐PD Consortium, Thomas Foltynie, Camille B Carroll, Roger Barker, James Carpenter, Yoav Ben‐Shlomo, Mark Edwards, Alan Whone, Carl Counsell, Caroline S Clarke, Matthew Burnell, Kate Hockey, Anna Jewell, Priti Gros, Tom Barber, Anette Schrag, Rimona S Weil, Caroline H Williams‐Gray, Michele T Hu, Lynn Rochester, Paola Piccini, Henrik Zetterberg, Alastair Noyce, Michael Lawton, Ashwani Jha, Brook Huxford, Shlomi Haar Millo, K. Ray Chaudhuri, Carroll Siu, Michèle Bartlett, Kuhan Pushparatnam, Daniel van Wamelen, Anthony HV Schapira, Oliver Bandmann, Simon Stott, George Tofaris, Esther Sammler, Heather Mortiboys, Li Wei, Alan Wong, Susan Duty, David Dexter, Edwin Jabbari, Stephen Mullin, Huw Morris, David Breen, Christian Lambert, Prasad Korlipara, Monty Silverdale, Kailash Bhatia, Alison Yarnall, Raj Khengar, Helen Collins, Fleur Hudson, Rebecca Croucher, Sandra Bartolomeu‐Pires, Veena Agarwal, Jennifer Allison, Jodie Forbes, Alex Edwards, Sheila Wonnacott, Dilan Athauda, Joy Duffen, Sonia Gandhi, Emily Henderson, Jen Black, Karen Matthews, Vince Greaves, Eric Deeson, Laurel Miller, Joel Handley, Helen Matthews, Kevin McFarthing, Amit Batla, Nikul Bakshi, Miriam Parry, Natasha Ratcliffe, Cheney Drew, Naveena Kapur, Anaya Navangul, Shafaq Ali, Katherine Fletcher, Claire Bale, Cristina Gonzalez‐Robles, Marie‐Louise Zeissler, Georgia Mills, Romy Ellis‐Doyle, Sally Collins, Rebecca Petty

**Affiliations:** ^1^ Department of Clinical and Movement Neurosciences, Queen Square Institute of Neurology University College London London United Kingdom; ^2^ John van Geest Centre for Brain Repair, Department of Clinical Neurosciences University of Cambridge Cambridge United Kingdom; ^3^ Parkinson's UK London United Kingdom; ^4^ Wolfson Sensory, Pain and Regeneration Centre, Institute of Psychiatry, Psychology & Neuroscience King's College London London United Kingdom; ^5^ North Bristol NHS Trust Bristol United Kingdom; ^6^ Expert Through Experience London United Kingdom; ^7^ Sheffield Institute for Translational Neuroscience, School of Medicine and Population Health The University of Sheffield Sheffield United Kingdom; ^8^ Faculty of Health University of Plymouth Plymouth United Kingdom; ^9^ Medical Research Council Protein Phosphorylation and Ubiquitylation Unit, School of Life Sciences University of Dundee Dundee United Kingdom; ^10^ Division of Neuroscience, School of Medicine University of Dundee Dundee United Kingdom; ^11^ Cure Parkinson's London United Kingdom; ^12^ Nuffield Department of Clinical Neurosciences University of Oxford Oxford United Kingdom; ^13^ Research Department of Practice and Policy, School of Pharmacy University College London London United Kingdom; ^14^ Pharmacy Service Royal Free Hospital NHS Foundation Trust London United Kingdom; ^15^ Translational and Clinical Research Institute Clinical Ageing Research Unit Newcastle University Newcastle United Kingdom

**Keywords:** Parkinson's disease, neuroprotective agents, neuroprotection, methodology, adaptive clinical trial

## Abstract

**Background:**

There are currently no disease‐modifying therapies (DMTs) registered for Parkinson's disease (PD). The Edmond J. Safra Accelerating Clinical Trials in Parkinson Disease (EJS ACT‐PD) initiative will expedite clinical assessment of putative DMTs through a multi‐arm multistage (MAMS) trial, testing several treatments against a common placebo arm and replacing unsuccessful therapies early.

**Objective:**

The objective of this study was to describe the treatment selection process for the EJS ACT‐PD clinical trial platform.

**Methods:**

A Treatment Selection Working Group (TSWG) identified compounds using complementary strategies, such as literature search, related initiatives (Cure Parkinson's International Linked Clinical Trials [iLCT] initiative), and expert suggestions. Compounds were classified into five mechanistic subgroups (mitochondrial, lysosomal, protein, inflammation, “other”). “Go/No‐Go” criteria and a scoring system covering preclinical, pharmacological, and clinical evidence were devised. Experts scored the candidates for quantitative rankings. Dossiers adapted from iLCT documents were produced for the top‐ranked compounds and in turn prioritized by the TSWG. Practical and logistical considerations from the Steering Committee (SC) guided the final decision. Patient and Public Involvement and Engagement representatives provided feedback throughout the process.

**Results:**

A total of 293 interventions were identified, 52 of which passed the “Go/No‐Go” criteria and were scored. Dossiers of the 14 top‐ranked compounds were submitted to the SC. Telmisartan, terazosin, and ursodeoxycholic acid were selected as the initial interventions.

**Conclusions:**

Drug selection in DMT PD MAMS trials requires consideration of scientific and practical issues. We present a robust system that can inform similar initiatives. © 2025 The Author(s). *Movement Disorders* published by Wiley Periodicals LLC on behalf of International Parkinson and Movement Disorder Society.

## Introduction

There are still no disease‐modifying therapies (DMTs) registered for PD. One contributing factor is the current inefficient classical trial designs to assess selected DMTs in PD. To address this, multi‐arm multistage clinical trials (MAMS) have been suggested as an alternative to traditional two‐arm designs.^1^ MAMS designs have been in use for years in other medical fields, such as oncology^2^ and virology,^3^, ^4^ and have already been implemented in other neurological disorders, namely, motor neuron disease (MND)^5^ and progressive multiple sclerosis (MS).^6^ In oncology, the Systemic Therapy for Advancing or Metastatic Prostate Cancer (STAMPEDE) MAMS trial, which started in 2005, has led to several improvements in standard of care and an increased survival in these patients.^2^, ^7^, ^8^, ^9^ As opposed to traditional two‐arm trial designs, MAMS trials test several treatments simultaneously against a common placebo arm. Early interim analyses are performed to allow for timely cessation of treatment arms without evidence of activity and their replacement with other promising treatments.^10^ MAMS trials have similarities with other designs already implemented in PD, mainly the early assessment of treatment arms, which was initially tested in PD via futility trials by the National Institute of Neurological Disorders and Stroke Neuroprotective Exploratory Trials in Parkinson Disease (NINDS NET‐PD) initiative, with the Futility Study 1 (FS‐1)^11^ and the FS‐TOO^12^ trials. The advantages of the MAMS trial design include sustained infrastructure, faster throughput of therapies, and a reduced proportion of participants in the placebo arm; its rationale in PD has been discussed elsewhere.^1^ Details of the EJS ACT‐PD trial design will be discussed in a separate publication (Zeissler et al, in preparation), but briefly, the EJS ACT‐PD trial is a UK‐wide study, assessing multiple putative DMTs for patients with PD aged 30 years or older without dementia and on stable dopaminergic medication in parallel against a shared placebo arm.

Scientifically robust, unbiased selection of the most promising disease‐modifying compounds is particularly important for MAMS trials, which are characterized by a faster throughput of compounds, requiring a trial‐ready list of suitable compounds at any point of the trial progression. This article describes the development and implementation of the treatment selection process for the Edmond J. Safra Accelerating Clinical Trials in Parkinson Disease (EJS ACT‐PD) MAMS trial of putative DMTs in PD.

## Materials and Methods

**FIG 1:**
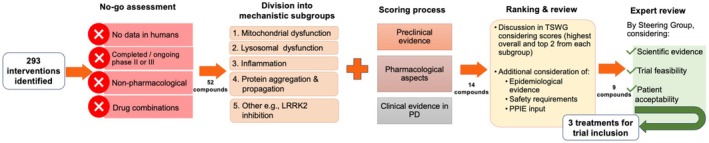
Simplified flowchart of the Edmond J. Safra Accelerating Clinical Trials in Parkinson Disease (EJS ACT‐PD) treatment selection process. LRRK2, leucine‐rich repeat kinase‐2; PD, Parkinson's disease; PPIE, Patient and Public Involvement and Engagement; TSWG, Treatment Selection Working Group. [Color figure can be viewed at wileyonlinelibrary.com]

The EJS ACT‐PD treatment selection process is a multistep system to identify the most promising putative DMTs for a MAMS trial in PD. It was developed and implemented between October 2021 and April 2023. An overview of the process is available in Supporting Information Figure S1, and the steps are briefly shown in Figure 1 and described in detail later. The process was undertaken by a Treatment Selection Working Group (TSWG) chaired by two of the authors (A.H.V.S. and O.B.) and comprising 13 additional members, including experts in preclinical and clinical aspects of PD, pharmacology, and four Patient and Public Involvement and Engagement (PPIE) members, with additional support from the Consortium's core team (co‐leads, clinical research fellow, research assistants, and administrators).

### Identification of Candidate Compounds

A range of complementary sources was used for identification of potential DMTs for EJS ACT‐PD. First, the nonconfidential dossiers for compounds evaluated by the International Linked Clinical Trials (iLCT) initiative, an annual collaborative clinical trial program for compound prioritization in PD, in the previous 10 years (2012–2022), kindly shared by Cure Parkinson's, were reviewed. In addition, the most up‐to‐date version of Dr. Kevin McFarthing's Hope List—a dataset of compounds being evaluated both preclinically and clinically in PD, which is reviewed and updated monthly—dated December 2021 was consulted,^13^ and the latest versions of an annual report describing the clinical trial pipeline for PD based on the ClinicalTrials.gov database^14^, ^15^ were also reviewed.

Furthermore, the dataset from a scoping review on DMT PD trials,^16^ shared by its first author, was reviewed, and all included interventions were listed for consideration.

A literature search was carried out in PubMed in May 2022, using the key words “Parkinson's disease” and “disease‐modifying,” focusing on clinical trials and articles in English. Where considered appropriate, bibliographic references in the retrieved articles were reviewed.

Lastly, suggestions of potential treatment candidates were invited from experts in PD and other neurodegenerative conditions with shared pathophysiology—multiple system atrophy, progressive supranuclear palsy, Huntington's disease, MND, and dementia—within and outside the EJS ACT‐PD Consortium.

### Compound Longlist

The interventions identified as part of the initial review were assessed against a predefined set of “Go/No‐Go” criteria (Table 1, Supporting Information Table S1) to obtain a longlist of compounds for subsequent scoring and ranking. In brief, only compounds with preclinical evidence or early clinical evidence of a potential disease‐modifying effect in PD and peer‐reviewed data in humans were included. Compounds in ongoing or recently completed trials were kept in a separate list for future review once the results of these trials are available. Compounds that had been assessed in clinical trials more than 5 years ago, typically identified on ClinicalTrials.Gov, but without any subsequent published results were excluded.

**TABLE 1:** Summary of inclusion and exclusion criteria for candidate interventions

Inclusion criteria	Exclusion criteria
✓ Disease‐modifying effect and/or rationale in PD ✓ Data in humans (at least phase 1 studies) ✓ Pharmacological interventions	✗ Negative phase 2 or 3 trials^a^ ✗ Nonpharmacological interventions (eg, gene therapy, cell therapy, exercise, devices, acupuncture, physiotherapy, aromatherapy) ✗ Disease‐modifying drug combinations (combinations of nutraceuticals are allowed [eg, Neuroaspis PLP10])

^a^ If negative phase 2 trial, consider the drug only if any significant change in dose from phase 2 in a new phase 2 or 3 study suggested, with rationale for dose‐dependent efficacy. A comment in the discussion of a paper is not enough, there must be rationale for a redesign (eg, one dose shows effectiveness and another one does not).

Abbreviation: PD, Parkinson disease.

Compounds were grouped according to their main mechanism of action: mitochondrial dysfunction, lysosomal dysfunction, inflammation, protein aggregation/propagation (α‐synuclein), and “other” (eg, leucine‐rich repeat kinase‐2 [LRRK2] inhibition). This was implemented to facilitate scoring by clinicians and scientists with particular expertise in the respective areas and with a view to combine different mechanistic effects in future trial arms via compound combinations with complementary modes of action.

### Longlist Scoring and Ranking

#### Compound Scoring System

A scoring system was developed by the TSWG and transferred to an online survey using the Research Electronic Data Capture (REDCap) software^17^, ^18^ (Supporting Information Table S2), for collaborators to score the compounds. In short, it comprised three sections (Preclinical Aspects, Basic Pharmacological Aspects, and Data from Previous Clinical Trials in PD), each of which had to be scored on a fixed scale: 0, 2, 6, or 10 points per section, thus enabling clear identification of front‐runners more easily than with a continuous scale. Therefore, the minimum score for a compound was zero, and the maximum was 30.

#### Scoring Process

To account for biases, at least three different experts in PD basic and clinical research from within the EJS ACT‐PD Consortium, including TSWG members plus nine additional experts, scored each compound independently. Scoring was aided by a spreadsheet comprising preclinical, pharmacological, and PD clinical data for each compound. Notably, the two cochairs of the TSWG (A.H.V.S. and O.B.) did not participate in the scoring to further reduce bias, and conflicts of interest were collated to identify any potential bias and remove or reallocate scoring as needed.

The average of the total score for each compound was calculated, and compounds were ranked according to their average scores, to produce a list of the top‐scoring compounds, including at least two compounds from each mechanistic subgroup. Each of the top‐ranking compounds was presented by one of its scorers at TSWG meetings to which all scorers and TSWG members, including PPIE representatives, were invited. An initial shortlist of compounds was compiled, including consideration of the strength of preclinical and clinical evidence, translatability to EJS ACT‐PD's target population, and side‐effect profile.

#### Treatment Dossiers

Detailed dossiers were created for the shortlisted compounds, following the structure of the iLCT nonconfidential dossiers. Additional searches and expert input allowed to update and finalize the remaining dossiers. The dossiers included the following sections: (1) summary of the intervention in PD, (2) clinical evidence in PD, (3) clinical evidence in other conditions (with a focus on neurodegenerative conditions), (4) epidemiological evidence, (5) pharmacology (safety, side effects, interactions, contraindications, etc.), (6) practical considerations—pharmaceutical, (7) practical considerations—funding, and (8) preclinical evidence (PD and other neurological conditions). Information to create the dossiers was obtained from the iLCT original dossiers, internet searches (PubMed, ClinicalTrials.gov, British National Formulary, European Medicines Agency, US Food and Drug Administration, and expert input from TSWG and Consortium members (eg, Funding Working Group [WG]).

### Decision on Initial Compounds

Dossiers for the shortlisted compounds were shared with the Steering Committee (SC) and PPIE representatives in advance of a 3‐day protocol writing meeting, which took place in December 2022. During this event, the shortlisted compounds were presented by the TSWG to the attendees and discussed (including consideration of logistical aspects of drug procurement and cost) before the final selection of the first three treatment arms of the EJS ACT‐PD MAMS trial platform.

### PPIE Input

Lay summaries of the shortlisted compounds were provided for the PPIE WG. These focused on aspects particularly relevant to people with PD (PwP), namely, mode of action, clinical experience, dose (including number of tablets/capsules per day and number of times they needed to be taken), interaction with other drugs/food, side effects, contraindications/cautions, monitoring required, and additional comments, where relevant. A dedicated meeting to review and discuss highly ranked candidate compounds with the PPIE WG was held in Spring 2023.

## Results

Details about the exact number of identified and prioritized compounds at each stage of the selection process can be found in Supporting Information Figure S2. In brief, from an initial list of 293 candidate interventions (198 after removing duplicates ‐ see Supporting Information Table S3), 63 overcame the “Go/No‐Go” criteria stage. These were initially reviewed by the TSWG Chairs and the trial co‐leads. This review led to 11 compounds being deselected at this stage for practical reasons, such as lack of availability due to commercial restraints, incompatibility with the EJS ACT‐PD MAMS trial design, which involves a UK‐wide large sample size (eg, infusions of fresh frozen plasma from young adults^19^), or in view of recent evidence suggesting that they would be unlikely to be effective as DMTs for PD.

The remaining 52 compounds were scored and ranked with the premise that at least two compounds should be considered for each mechanistic subgroup. The classification of the shortlisted 14 compounds was as follows: two “mitochondrial,” two “lysosomal,” two “inflammation,” two “protein aggregation/propagation,” and six “other.”

Discussion of those 14 compounds within the TSWG, factoring in preclinical, clinical, and pharmacological evidence, as well as epidemiological data, safety considerations, and PPIE input, resulted in a final shortlist of 9 compounds, for which dossiers were prepared. After review of the dossiers and discussion with the SC, considering scientific evidence, trial feasibility, and patient acceptability, three compounds were initially selected for inclusion in the trial (telmisartan, istradefylline, and ursodeoxycholic acid [UDCA]); one was deemed to require review of its evidence and was therefore put in a “reserve list” (lovastatin), and five were removed from the selection process (fasudil, levetiracetam, D‐serine, fingolimod, and cGPMAX, a cyclic Glycine‐Proline supplement). The limited number of promising, available compounds prompted a request for further suggestions to the SC and the International Advisory Group. At this point, a previous key “Go/No‐Go” criterion was also changed, namely, the exclusion of compounds with ongoing or planned phase 2/3 trials, to assess their neuroprotective potential further, because the trials’ remits may differ and be complementary rather than redundant. After reviewing early‐phase evidence of clinical efficacy, as well as previous trial results, where available, additional consideration was given to the following nine compounds: AZD3241/Verdiperstat, CNM‐Au8, dl‐3‐n‐butylphthalide, isradipine controlled release, Neuroaspis PLP10, terazosin, vinpocetine, probiotic supplements, and deferiprone, as well as lovastatin, from the initial nine shortlisted compounds. Scoring of these 10 additional compounds by the TSWG (applying the same criteria as before) and review of previous scores of the initial nine shortlisted compounds led to the following final shortlist of eight compounds, in descending order of scores: telmisartan, terazosin, UDCA, istradefylline, lovastatin, deferiprone, Neuroaspis PLP10, and AZD3241. The first three compounds to be included in the trial will therefore be telmisartan, terazosin, and at a later time point, UDCA. Details on these three compounds and rationale for their prioritization is included in Table 2.

**TABLE 2:** Initial three compounds and evidence supporting their inclusion in the trial

	Telmisartan	Terazosin	UDCA
Brief description	ARB licensed as an antihypertensive drug Antiinflammatory and overall neuroprotective effects through various mechanisms, such as PPAR‐γ activation	α1‐AR antagonist, licensed for the treatment of hypertension and benign prostatic hyperplasia Activates PGK1, a central glycolysis enzyme, enhancing ATP production	Naturally occurring bile acid In clinical use for >30 years for the treatment of primary biliary cholangitis Restores mitochondrial function through multiple mechanisms
Preclinical evidence	Neuronal cell models: *SK‐N‐SH human neuroblasts and primary rat cortical neurons*: Amelioration of IL‐1β–induced neuronal inflammatory response more potently than similar BBB‐penetrant ARBs, candesartan and losartan^1^	Neuronal cell models: *MPP‐treated M17 human neuroblastoma cells*: Enhancement of glycolysis (increased pyruvate levels, citrate synthase activity, and ATP levels)^2^	Neuronal cell models: *SH‐SY5Y human dopaminergic cells*: Amelioration of sodium‐nitroprusside–induced cytotoxicity with attenuation of ROS production, inhibition of both mitochondrial membrane potential loss and intracellular reduced GSH depletion^3^ *Mouse cortical neurons*: Rescue of cellular ATP levels in mouse cortical neurons with siRNA‐mediated *parkin* knockdown^4^ *Mouse neuroblastoma (Neuro‐2a) cells*: Improved cell viability and decreased cell death; inhibition of ROS accumulation and improved mitochondrial function, as well as amelioration of autophagic flux in MPP^+^‐treated cells^5^ *Mesencephalic dopaminergic C57BL/6 mouse neurons*: Normalization of anterograde and retrograde mitochondrial transport^5^
	Animal models: *MPTP mouse models*: Inhibition of MPTP‐induced microglial/astroglial response (↑ BDNF, GDNF, GSH; ↓ GFAP)^6‐8^ Improvement of mitochondrial function (↑ PINK1, Parkin, LRRK2, DJ‐1, MTA1 UCHL1)^9^ Amelioration of dopaminergic function (↑ TH, DAT, VMAT2; ↓ α‐syn)^7,8^ Recovery of locomotor performance^7‐9^ *α‐Syn (SynA53T) rat model* ^10^: α‐Syn overexpression: ↑ AT1R expression ↑ Proinflammatory/M1 microglial phenotype ↓ Immunoregulatory/M2 microglial phenotype ↑ α‐syn–induced dopaminergic neuron death TEL: amelioration/inhibition of the above *Rotenone rat model* ^11^: ↓ ER stress–mediated apoptosis (↓ IRE1α‐TRAF2‐caspase‐12 pathway, ↑ PPAR‐β/δ activation) ↓ α‐Syn accumulation, dopamine depletion Symptomatic improvement	Animal models: *MPTP mouse models*: Increase in brain ATP levels, enhancement of glycolysis, and increased mitochondrial DNA levels^2^ Reduction in TH‐positive neuron death and improvement in dopaminergic function (attenuated decrease of striatal and nigral DOPAC and HVA content)^2^ *6‐OHDA mouse models*: Motor function improvement and attenuation of dopaminergic neuron degeneration (increase in DOPAC and HVA content)^2^ *PINK1* ^*5*^ Drosophila *fly model*: Partial reversal of motor deficits^2^ *LRRK* ^*ex1*^ Drosophila *fly model*: Amelioration of motor deficits^2^ *Rotenone* Drosophila *fly model*: Attenuation of decrease in ATP levels and of deterioration of motor performance^2^ Lack of the above effects in the rotenone‐treated PGK1 knockdown *Drosophila* model^2^ Resistance to rotenone‐induced behavioral alterations on *Drosophila* flies overexpressing PGK1^2^	Animal models: *LRKK2G2019S transgenic flies*: Marked improvement of contrast response function (vision) at all three neuronal levels (photoreceptors, lamina neurons, and medulla neurons)^12^ *MPTP‐treated mice*: Improved motor performance, increase in TH‐positive neurons in the SNpc and TH‐positive fibers in the striatum^5^ *Rotenone rat model*: Improved motor performance, normalization of striatal dopamine content, reduction in striatal cytokine expression (TNF‐α, IL‐1β, IL‐10), elevation of striatal ATP levels, and preservation of mitochondrial integrity^13^
	Human and monkey tissue^14^: Major RAS components present in dopaminergic neurons, astrocytes, and microglia in SNc of both humans and monkeys Human tissue: Dopaminergic neuron dysfunction/loss related to ATII/AT1/Nox4 axis‐mediated oxidative stress, suggesting ↓ total and nuclear AT1 expression ↑ neuron survival^15^ High susceptibility to PD of the dopaminergic nigral cell subpopulation expressing the AT1R gene^16^	Human tissue: *iPSC‐derived neurons from LRRK2* ^*G2019S*^ *‐related PD*: Increase in ATP levels and reduction of α‐syn accumulation^2^	Human tissue: Marked mitochondrial rescue effect in *parkin*‐mutant and *LRRK2* ^*G2019S*^ mutant (PD patient and asymptomatic carrier) fibroblasts, as well as in mechanistically stratified fibroblasts from sporadic PD patients^17^
Target engagement and CNS penetration	TEL: greatest CNS penetrance (high lipophilicity) and most favorable pharmacokinetics of all ARBs^18‐20^ Established PK serum profile in humans: single dose 40 mg orally^21^ Multiple animal studies showing central actions of peripherally administered TEL and other ARBs (see previous section) CNS penetration demonstrated in PET scans in macaque monkeys^22^ No formal human studies of CNS penetration; however, consensus that TEL (and ARBs) act centrally and metaanalyses/epidemiological studies pointing to central effects of TEL and other ARBs^23‐25^	High bioavailability and lipophilicity Effects in animal models indicate brain penetration (see previous section) CNS penetration demonstrated in a placebo‐controlled pilot study in PD patients (TZ 5 mg/day): ↑ ßATP/inorganic phosphate brain ratio on 31P‐MRS, ↑ blood ATP levels^26^ Dose‐finding study: oral TZ 5 mg/day achieves effective target engagement^27^	Established PK serum profile in PD patients^28^ Pilot PK study in PD patients showed increase in brain ATP and decrease in brain ATPase activity on 31P‐MRS^28^ Confirmed CSF penetrance at higher doses (30–45 mg/kg) in patients with motor neuron disease^29^ 31P‐MRS evidence of midbrain target engagement in a phase 2, randomized, double‐blind, placebo‐controlled trial (“UP” study)^30^
Epidemiological evidence	Reduced risk of PD among ARB users,^31^ especially with brain‐penetrant ARBs,^32^ such as telmisartan AT1R‐AA higher in PD patients versus controls^33^ Increased levels of neuroinflammation markers, inhibited by a similar drug, candesartan^33^ ARB use associated with verbal memory improvement at 3 years^23,24^	Reduced incidence and milder progression of PD among people taking terazosin and other PGK1 activators versus α1‐AR antagonists, which do not activate PGK1^2,34‐36^	Multiple reports of bile acid abnormalities in PD^37,38^
Clinical evidence (PD)	Small trial of candesartan in patients with PD and hypertension showed good tolerability and a significant clinical improvement (HAD, UPDRS I‐IV, PDQ‐39)^39^ Various ongoing trials on ARB in other neurodegenerative conditions, such as AD	Pilot study to assess target engagement of terazosin in PD (8 patients on TZ, 5 on placebo, 12‐week treatment duration): 3/8 (37.5%) dropout in TZ treatment arm due to orthostatic hypotension‐related symptoms; increase of brain βATP to inorganic phosphate ratio in the brain (31P‐MRS) and of blood ATP levels versus placebo group^26^	Phase 2 “proof‐of‐concept” study (“UP” study, 30 patients with 2:1 split UDCA 30 mg/kg vs. placebo, treatment over 48 weeks)^30^: Excellent safety and tolerability (primary outcome) and tentative improvement of gait in supervised gait analysis (secondary outcomes) A high mitochondrial polygenic risk score predicts the response to UDCA vs. placebo (as quantified by 31P‐MRS)^40^
Practical considerations	Best pharmacokinetics of all ARBs Long‐term experience of use Excellent side‐effect profile Most relevant side effect in PD: orthostatic hypotension, which has been factored into the trial's design to ensure participant safety Oral once‐daily tablet	Except for the risk of orthostatic hypotension, terazosin is well tolerated in general Long‐term experience of use Most relevant side effect in PD: orthostatic hypotension, which has been factored into the trial's design to ensure participant safety Oral once‐daily tablet	>95% compliance in UP study Most common side effect: transient, mild loose stools Patients will typically need to take a total of 4–5 tablets (500 mg each) per day in three doses The selected formulation (Ursofalk 500 mg tablets) is coated, scored, and of similar size to standard PD medication

*Note*: References 1–40 are available in the Supporting Information.

Abbreviations: UDCA, ursodeoxycholic acid; ARB, angiotensin II type 1 receptor blocker; PPAR, peroxisome proliferator‐activated receptor; α1‐AR, α1‐adrenergic receptor; PGK1, phosphoglycerate kinase 1; ATP, adenosine triphosphate; SK‐N‐SH, human neuroblastoma cell line; IL‐1β, interleukin‐1β; BBB, blood–brain barrier; MPP, 1‐methyl‐4‐phenylpyridinium; ROS, reactive oxygen species; GSH, glutathione; MPTP, 1‐methyl‐4‐phenyl‐1,2,3,6‐tetrahydropyridine; BDNF, brain‐derived neurotrophic factor; GDNF, glial cell line–derived neurotrophic factor; GFAP, glial fibrillary acidic protein; PINK1, PTEN induced putative kinase 1; LRRK2, leucine‐rich repeat kinase‐2; DJ‐1, Parkinson Disease Protein 7; MTA1, Metastasis‐Associated (protein) 1; UCHL1, Ubiquitin Carboxy‐Terminal Hydrolase L1; TH, tyrosine hydroxylase; DAT, dopamine transporter; VMAT2, vesicular monoamine transporter 2; α‐syn, alpha‐synuclein; SynA53T, human alpha‐synuclein, A53T variant; AT1R, angiotensin type 1 receptor; TEL, telmisartan; ER, endoplasmic reticulum; IRE1α, inositol‐requiring enzyme 1 alpha; TRAF2, tumor necrosis factor receptor–associated factor 2; DOPAC, 3,4‐dihydroxyphenylacetic acid; HVA, homovanillic acid; 6‐OHDA, 6‐Hydroxydopamine; TNF‐α, tumor necrosis factor alpha; RAS, renin‐angiotensin system; SNc, substantia nigra, pars compacta; ATII, angiotensin II; AT1, angiotensin 1; Nox4, Nicotinamide Adenine Dinucleotide Phosphate Oxidase 4; PD, Parkinson's disease; iPSC, induced pluripotential stem cell; CNS, central nervous system; PET, positron emission tomography; PK, pharmacokinetics; TZ, terazosin; 31P‐MRS, 31‐phosphorus magnetic resonance spectroscopy; CSF, cerebrospinal fluid; AA, autoantibodies; HAD, Hospital Anxiety and Depression scale; UPDRS, Unified Parkinson's Disease Rating Scale; PDQ‐39, 39‐item Parkinson's Disease Questionnaire; AD, Alzheimer's disease; SNpc, substantia nigra pars compacta.

## Discussion

Previous neurological MAMS trials have employed different approaches toward compound identification and prioritization. The Motor Neuron Disease Systematic Multi‐Arm Adaptive Randomized Trial (MND‐SMART), a MAMS trial in MND,^5^ developed a two‐stage systematic review of potential DMTs aided by machine learning (ML) and text‐mining techniques. In stage one, clinical studies in different neurological conditions, including MND, were initially reviewed and scored according to safety, efficacy, study size, and study quality. In the second stage, the authors reviewed the efficacy of drugs in cell and animal models of MND, including human induced pluripotent stem cell studies. Two shortlisting rounds and a final selection round were carried out by a panel of experts, taking into consideration the findings from the systematic review, late‐breaking evidence, mechanistic plausibility, safety, tolerability, and feasibility of evaluation in the MND‐SMART trial.^20^

Drug selection for the Optimal Clinical Trials Platform for Multiple Sclerosis (OCTOPUS), a MAMS trial in progressive MS, consisted of an initial systematic review of clinical studies of oral already‐licensed putative DMTs in MS and other neurological conditions with shared pathophysiology, followed by a systematic review and meta‐analysis of the available in vivo experimental evidence for the identified compounds. An international expert committee reviewed the compound list and assessed the potential candidates against a set of prespecified criteria, leading to identification of the compounds to be included in the first iteration of the trial.^21^

Regarding other DMT prioritization efforts, the iLCT initiative is a collaborative clinical trial program led by Cure Parkinson's and the Van Andel Institute. Literature reviews and discussions with pharmaceutical companies produce an initial list of potential DMTs in PD, which then undergo a comprehensive assessment—mechanism of action, clinical and preclinical data, and safety. Detailed dossiers are produced for the most promising compounds. These are reviewed by a multiprofessional committee of PD experts ahead of their annual 2‐day meeting, during which the compounds are scored and the top‐ranking therapeutics are prioritized for clinical evaluation.^22^, ^23^ Nonconfidential iLCT dossiers of the prioritized compounds from the last 10 years were kindly shared by Cure Parkinson's, and their structure helped create this group's compound dossiers.

A further useful resource for monitoring potential candidates for treatment selection is Dr. Kevin McFarthing's “Hope List,”^13^ which is a monthly reviewed registry of preclinical and clinical therapies under development for PD, both symptomatic and disease modifying. Dr. McFarthing also leads an annual review of agents in clinical trials for PD that was initiated in 2020, has been published every year since,^14^, ^15^, ^24^, ^25^ and is an invaluable, freely available resource for researchers, clinicians, and PwP.

The earlier‐described selection process was iterative and pragmatic. It benefitted considerably from already existing previous efforts to identify and prioritize promising DMTs. Moreover, the “Go/No‐Go” criteria and the scoring system were extensively reviewed and repeatedly revised by the expert members of the TSWG, PPIE members, international colleagues, and the wider EJS ACT‐PD Consortium.

Due to different posology (eg, number of doses per day) or form of administration (eg, oral versus intravenous), a shared placebo arm may not be feasible across all EJS‐ACT‐PD treatment arms.

In those cases, the placebo allocation ratio may vary, which, together with other factors, can impact the participant's expectation of benefit and result in differential placebo effects, an issue already reviewed in PD.^26^ For EJS‐ACT‐PD, the first two active arms are oral tablets taken once daily, allowing for a common placebo arm, but the upcoming UDCA trial arm will require a separate placebo arm initially because of its different posology. To address this, careful consideration has been given to trial design (Zeissler et al, in preparation).

The current treatment selection process and criteria will be regularly reviewed and refined. Currently planned treatment arms include only repurposed compounds, but commercial entities and academics will be encouraged to approach the TSWG with candidate compounds for potential inclusion as future treatment arms. Annual literature reviews will be performed to update the evidence on existing compounds and to identify new candidates. The application of artificial intelligence/ML approaches to these reviews, such as in the drug selection process for the MND‐SMART trial,^20^ is an attractive idea but is not expected to be implemented in the EJS ACT‐PD trial in the immediate future.

Future drug selection processes may also include a preclinical “in‐house” component (“trial in a dish”) and a “derisking” phase 2 MAMS platform that could assess promising compounds for their pharmacokinetic properties and confirmation of target engagement. Collaboration with similar international initiatives (ie, the Path to Prevention [P2P] platform trial in the United States,^27^ the NS‐Park master trial in France,^28^ the Australian Parkinson's Mission in Australia,^29^ and the SLEIPNIR trial in Norway^30^) will be considered.

### Conclusions

Treatment selection for disease‐modifying trials in PD is a challenging, yet crucial part of the quest for DMTs in this condition. We present a thorough, multistep process that will hopefully inform similar initiatives in other fields.

## Author Roles

(1) Research project: A. Conception, B. Organization, C. Execution. (2) Statistical Analysis: A. Design, B. Execution, C. Review and Critique. (3) Manuscript Preparation: A. Writing of the First Draft, B. Review and Critique.

C.G.‐R.: 1A, 1B, 1C, 3A, 3B

D.A.: 1C, 3B

T.R.B.: 1C, 3B

R.A.B.: 1C, 3B

D.T.D.: 1B, 1C, 3B

S.D.: 1B, 1C, 3B

R.E.‐D.: 1B, 1C, 3B

S.G.: 1C, 3B

J.H.: 1C, 3B

E.J.: 1B, 1C, 3B

K. Martin: 1B, 1C, 3B

K. McFarthing: 1B, 1C, 3B

G.M.: 1B, 1C, 3B

H.M.: 1B, 1C, 3B

S.M.: 1C, 3B

R.P.: 1B, 1C, 3B

E.S.: 1B, 1C, 3B

P.S.: 1B, 1C, 3B

S.R.W.S.: 1B, 1C, 3B

G.K.T.: 1B, 1C, 3B

L.W.: 1B, 1C, 3B

C.H.W.‐G.: 1C, 3B

A.W.: 1B, 1C, 3B

M.‐L.Z.: 1A, 1B, 1C, 3B

R.K.W.: 1A, 3B

C.B.C.: 1A, 1B, 1C, 3B

T.F.: 1A, 1B, 1C, 3B

O.B.: 1A, 1B, 1C, 3B

A.H.V.S.: 1A, 1B, 1C, 3B

## Financial Disclosures

C.G.‐R., R.E.‐D., G.M., R.P., and M.‐L.Z. have no conflict of interest to report and have received research support from the Edmond J. Safra (EJS) Foundation (EJS Accelerating Clinical Trials in Parkinson's Disease (EJS ACT‐PD) project). T.R.B., K. Martin, K. McFarthing, P.S., and A.W. have no conflict of interest nor financial disclosures to report. D.A. has no conflict of interest and receives funding from National Institute for Health and Care Research/Medical Research Council (NIHR/MRC) Clinical Academic Research Partnership. R.A.B. has received grants from the NIHR, The Michael J. Fox Foundation (MJFF), Cure Parkinson's Trust, Rosetrees Trust, EU, MRC, and Wellcome; has served on Advisory Boards for Living Cell Technologies, Aspen Neuroscience, BlueRock Therapeutics, Novo Nordisk, TreeFrog Therapeutics, Rinri Therapeutics, Harness, and UCB; has received honoraria for talks sponsored by Novo Nordisk; and receives royalties from Springer Nature and Wiley. S.D. has received funding from Parkinson's UK and serves on the Advisory Board of Nevrargenics Ltd; and has performed preclinical research on the protective potential of riluzole, but she was not involved in its scoring during this selection process. D.T.D. serves on the scientific Advisory Board for Herantis Pharma, Finland. S.G. has no conflicts of interest, is an MRC Senior Clinician Scientist, and is supported by an MRC Fellowship. J.H. has no conflict of interests to declare and is currently funded by a Parkinson's UK Fellowship via the Association of British Neurologists. E.J. has no conflict of interest to report and is supported by the PSP Association (PSPA2023/PROJECTGRANT001), CurePSP (681‐2022/06), and the MRC (548211). H.M. has received grants from Parkinson's UK, Cure Parkinson's, Alzheimer's Research UK, Rosetrees Trust, and MJFF, and has been involved in the development of UDCA. S.M. has no conflict of interest to report and is currently funded by the UKRI MRC (MR/Y503472/1). E.S. has received funding from UKRI MRC Core and the MJFF; has received consultancy payments from Bial; and has received financial support for attending meetings from Bial and AbbVie. S.R.W.S. has no conflicts beyond being an employee of Cure Parkinson's. G.K.T. has no conflict of interest to report and is funded by an MRC Senior Clinical Fellowship (MR/V007068/1). L.W. has received grants from National Institute of Health Research, Cure Parkinson's, Parkinson's UK, Diabetes UK, and Innovation and Technology Commission, Hong Kong. C.H.W.‐G. is supported by the MRC (MR/W029235/1) and the NIHR Cambridge Biomedical Research Centre (NIHR203312). The views expressed are those of the authors and not necessarily those of the NIHR or the Department of Health and Social Care. R.K.W. has no conflicts beyond being an employee of Cure Parkinson's. C.B.C. has received advisory, consulting, or lecture fees from AbbVie, Britannia, Mission Therapeutics, and Roche, and has received research funding from Parkinson's UK, EJS Foundation, NIHR, and Cure Parkinson's. T.F. has received grants from NIHR, EJS Foundation, MJFF, John Black Charitable Foundation, Cure Parkinson's, Parkinson's UK, Gatsby Foundation, Innovate UK, Janet Owens Research Fellowship, Rosetrees Trust, Van Andel Research Institute, and Defeat MSA; has served on Advisory Boards for Peptron, Voyager Therapeutics, Handl Therapeutics, Gain Therapeutics, Living Cell Technologies, AbbVie, BlueRock, Bayer, and Bial; and has received honoraria for talks sponsored by Bayer, Bial, Profile Pharma, Boston Scientific, and Novo Nordisk. O.B. has received grants from Cure Parkinson's (OB02), the MRC Centre of Excellence Award (CoEN) (MR/V006525/1), and the Jon Moulton Charity Trust; has served on the Advisory Board for ICE; and has been involved in the development of UDCA, but he was not involved in its scoring during this selection process. A.H.V.S. has received grants from the MRC (UK), Cure Parkinson's Trust, Parkinson's UK, EU H2020 program and Aligning Science Against Parkinson's, MJFF, and Innovate UK, and consults for Capsida and Neurocrine.

## Supporting information


**Data S1** Supporting Information.

## Data Availability

Data sharing is not applicable to this article as no new data were created or analyzed in this study.
