# Supplementary material for: Treatment Selection and Prioritization for the EJS ACT‐PD MAMS Trial Platform
Source: Mov Disord. 2025 Apr 18;40(7):1307–17. doi: 10.1002/mds.30190 (PMC12273612; doi:10.1002/mds.30190)
Supplement: Supplementary file 1 — Data S1 Supporting Information. [file MDS-40-1307-s001.zip › mds30190-sup-0004-Supp Mat – EJS ACT-PD treatment selection manuscript revised 2025.02.07_clean_corrected2025.04.07.docx]

**Treatment selection and prioritization for the EJS ACT-PD MAMS trial platform. Supplementary material**

Supplementary Figure 1. Detailed schematic of the EJS ACT-PD treatment selection process.
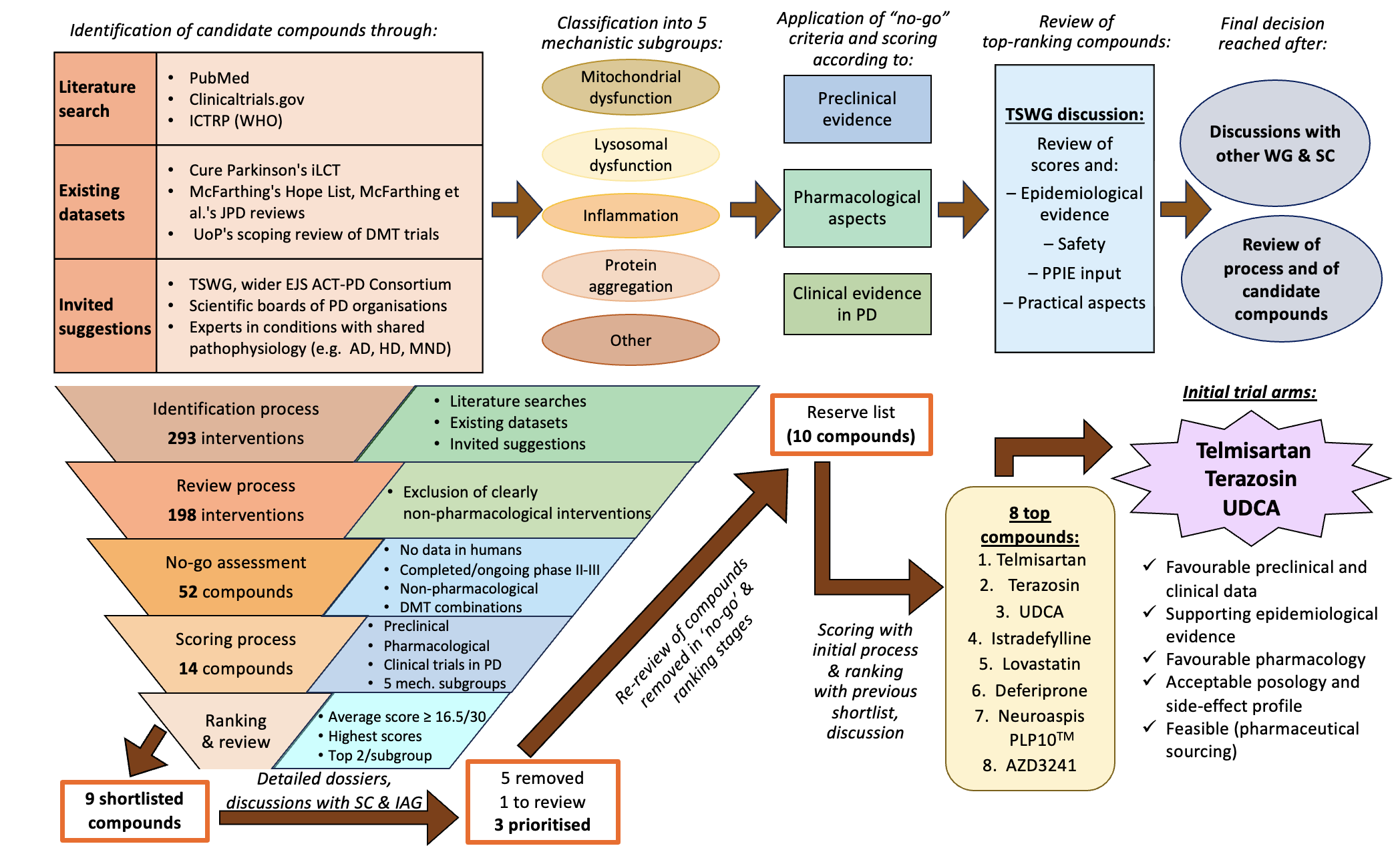


Abbreviations: AD: Alzheimer’s disease; DMT: disease-modifying therapy; EJS ACT-PD: Edmond J Safra Accelerating Clinical Trials in Parkinson’s Disease (initiative); HD: Huntington’s disease; IAG: International Advisory Group; CTRP: International Clinical Trials Registry Platform; iLCT: international Linked Clinical Trials (initiative); JPD: Journal of Parkinson’s Disease; mech.: mechanistic; MND: motor neuron disease; PD: Parkinson disease; PPIE: Patient and Public Involvement and Engagement; SC: Steering Committee; TSWG: Treatment Selection Working Group; UDCA: ursodeoxycholic acid; UoP: University of Plymouth; WG: Working Group(s); WHO: World Health Organization.

Supplementary Table 1. EJS ACT-PD Treatment Selection “No-Go” criteria decision tree.

| **Step** | **Checklist** | **Yes** | **No** |
| --- | --- | --- | --- |
| 1 | Are there any **data in humans** for this compound (including safety data (i.e. phase 1 studies))?^a^ | **Go to step 2**. All repurposed compounds (e.g. iLCT) have data in humans, by definition. | **Record & Exclude**  Also exclude compounds currently under phase 1 studies or with completed phase 1 studies with no published results^b^ – Track separately |
| 2 | Are there any **ongoing neuroprotective phase 2 or 3 trials** on this compound? ^c^ | **Record & Exclude** for now – Track in a separate list to review progress and when ongoing trials are completed, consider inclusion depending on results. | **Go to Step 3** |
| 3 | Are there any **completed neuroprotective phase 2 or 3 trials** on the compound? ^c^ | Document phase and result of trial: | **Go to Step 4** |
|  |  | Positive phase 2 trials **with** ongoing or planned phase 3 – **Record & Exclude** – Track separately to review progress and when ongoing trials are completed, consider inclusion depending on results.  Negative phase 2 or 3 trials – **Record & Exclude** (document link/details on negative trials to allow for future review if deemed appropriate). |  |
|  |  | If negative phase 2 trials 🡺 Search for post hoc analyses. If significant change in dose from phase 2 in a new phase 2 or 3 study, with rationale for dose-dependent efficacy 🡺 **Go to Step 4** |  |
|  |  | Positive phase 2 trials **without** ongoing or planned phase 3, and positive phase 3 trials 🡺 **Go to Step 4** |  |
| 4 | Are these **pharmacological interventions**, **including nutraceuticals** (but excluding supplements (probiotics, seeds))? ^d^ | If compounds from APM/iLCT/Hope List/UoP scoping review 🡺 **Go to step 6** | **Record & Exclude** – Document well (leave links/references) for potential future review |
|  |  | If not (i.e. compounds found by literature search or invited suggestions) **🡺 Go to step 5a** |  |
| 5a | Abstract/description/ title clearly states that the intent is to find evidence for **disease-modification/neuroprotection** of the intervention being investigated? | **Go to Step 6** | **Go to Step 5b** |
| 5b | Google search of NCT number and/or drug reveals its indication is for **managing symptoms on the first page of results** and there is **no statement** in abstract or title indicating that this drug is thought to **modify the disease course**? | **Record & Exclude** | **Go to Step 5c** |
| 5c | Google search of NCT number and/or drug reveals public statement that the trial intent is **disease-modifying on first page of results**? | **Go to step 6** | **Record & Exclude** – In future rounds, consider reviewing whether there is preclinical evidence of disease-modification for these compounds |
| 6 | **Drug combinations** (both/all compounds with **disease-modifying** potential)?^e^ | **Record & Exclude** – Consider including drug combinations in the future, when feasible | **Go to Step 7** |
| 7 | Is the compound **licensed** for any use (in the BNF)? | **Include in longlist** | **Keep in a separate list** |

Abbreviations: APM: Australian Parkinson’s Mission; BNF: British National Formulary; iLCT: international Linked Clinical Trials initiative; MAMS: multi-arm, multi-stage (clinical trial); NCT: National Clinical Trials (number); UoP: University of Plymouth.

^a^ Search compound in its original source database: either the Hope List, McFarthing et al. JPD 2021 (10.3233/JPD-219006), 2022 (10.3233/JPD-
 229002), or in University of Plymouth’s scoping review database

Search: (compound) in PubMed, with the “Clinical Trial” filter on, and review results

Search: [compound] in clinicaltrials.gov, with no additional filters, and review results

Search: [compound] in ICTRP (trialsearch.who.int), with no additional filters, and review results

Alternatively, or If no information in the above searches: Google: [compound] + [Parkinson], review 1st page of results (e.g. nilotinib Parkinson) – If there are 2 names for a compound, try both separately + Parkinson – If nothing comes up, try [compound] + “safety” (aim is to know if there are any safety data at all on that compound, not only in PD). Safety data must be in a publication, or the compound must have been progressed to phase 2. A simple press release/statement in a pharma website is not enough.

^b^ No results after searching PubMed, clinicaltrials.gov, and the first page of Google search of NCT number and/or drug.

^c^ Search: Parkinson + [compound] in clinicaltrials.gov, with no additional filters.

Search: Parkinson’s disease, [compound] in the International Clinical Trials Registry Platform (ICTRP) search portal
 (<https://trialsearch.who.int>), with no additional filters.

Search: (parkinson*) AND (compound), with the “Clinical Trial” filter on, and review results.

Search compound in Hope List, and McFarthing et al. 2021 (10.3233/JPD-219006) and 2022 (10.3233/JPD-229002).

Search compound in University of Plymouth’s scoping review database (doi:10.1136/bmjopen-2023-071641).

When no information in several of the above: Google (<https://www.google.com/>): [compound] + [PD] or [Parkinson], review first page of results.

^d^ Excluded: exercise, physiotherapy, aromatherapy, diet, surgical interventions, devices, gene therapy, cell therapy.

^e^ Combinations of a disease-modifying compound with a symptomatic compound should be included.

Supplementary Table 2. Scoring system for the EJS ACT-PD treatment selection process.

| Section 1: Preclinical Aspects – Consider the following: |
| --- |
| *Mechanism of action:*   - How many modes of action (1 vs more than 1) - How well defined is the mode of action (target/pathway-specific e.g. Akt activation) - What was the impact on pathophysiology (e.g. rescue of mitochondrial function)   *Reproducibility and nature/quality/relevance of model(s) used:*   - How many preclinical models was effect demonstrated in (1 vs more than 1)? - Was a beneficial effect of this compound only observed in vitro or in vivo or both in vitro and in vivo? - If more than one model - did the different preclinical models have complementary validity (e.g. in vitro and in vivo, in patient tissue and in animal models, modelling different aspects of the disease, such as alpha-synuclein propagation and mitochondrial dysfunction, etc.)? - Has an effect been demonstrated in an alpha-synuclein model? - Were all preclinical studies conducted by the same group or by two or more groups? - Were effects seen on pathology/pathway (e.g. Akt activation, alpha-syn reduction; TH-positive cell protection) only or were functional (e.g. motor/cognitive) improvements observed as well? - What is the translational potential of the preclinical model? Please consider the following when judging this:   Models closer to human PD (iPSC, etc.) should be considered more translatable than those further from it (e.g. animal toxin models):  Preclinical *in vitro* models – Cell models (higher to lower value):   - Specific neuronal models e.g. iPSCs from patients - Non-neuronal patient tissue (e.g. fibroblasts) - Primary cultures from genetically modified models e.g. mouse cortical neurons - Non-specific cultures e.g. HEK293, SHSY-5Y   Preclinical *in vivo* models – Animal models (higher to lower value):   - Non-human primates - Rodents - Other (e.g. zebrafish, *Drosophila*)   Models that are more suitable for the proposed mechanism of the particular drug could potentially be more useful/of greater translational value than models that are less suitable for a drug's mode of action (e.g. rotenone for a drug with an impact on mitochondrial function, an inflammation model for an anti-inflammatory drug). |
| Section 2: Basic Pharmacological Aspects – Consider the following: |
| - Blood brain barrier penetration (if required for action) – Also consider whether the drug reaches the CNS at a relevant dose and/or in an appropriate timeframe. - If BBB penetrance not required for action (e.g. Azathioprine), consider other relevant pharmacokinetic aspects (e.g. protein bound/free, effective dose likely to be achieved in human patients, etc.) - Confirmed target engagement (e.g. Akt activation) and/or functional effect (e.g. rescue of mitochondrial function) in animal models only - Confirmed target engagement and/or functional effect in humans |
| Section 3: Data from Previous Clinical Trials in PD – Consider the following: |
| - Was there only one clinical trial in PD or more than one?   - If more than one clinical trial in PD, were all trials carried out by the same group or different groups?   - Was the effect of this compound on clinical progression in PD assessed using clinical assessment only (typically MDS-UPDRS-III) or were additional tools used (e.g. DaT-SPECT, sensor-based objective quantification of motor impairment)? - Was the compound tested a repurposed compound or a novel compound? |

Abbreviations: Akt: protein kinase B; alpha-syn: alpha-synuclein; BBB: blood-brain barrier; CNS: central nervous system; DaT-SPECT: dopamine transporter single-photon emission computed tomography; HEK293: Human Embryonic Kidney 293; iPSC: induced pluripotent stem cells; MDS-UPDRS-III: Movement Disorders Society-sponsored revision of the Unified Parkinson’s Disease Rating Scale, part III; PD: Parkinson disease; SHSY-5Y: chromosomally stable human neuroblastoma cell line; TH: tyrosine hydroxylase.

Supplementary Table 3. List of the 198 compounds screened against Go/No-Go criteria.

| **Compounds screened against Go/No-Go criteria (198)** | |
| --- | --- |
| 1. ACI-7104 (PD01A) 2. AKST4290-211 3. AL101 4. AlloRx 5. Alogliptin 6. Alpha-dihydroergocryptine 7. Alpha-tocopherol and acorbate 8. Ambroxol 9. ANAVEX2-73 10. Anle 138b 11. ANVS-401 (Posiphen) 12. ATH-1017 (NDX-1017) 13. ATH434 (PBT434) 14. Atuzaginstat 15. Axitinib 16. Azathioprine 17. AZD3241 18. Bee venom 19. Benfotiamine 20. BIA28-6156/LTI-291 21. BIIB094 22. BIIB118 23. Bromocriptine 24. Bumetanide 25. Bushenpingchan formula 26. Butylphtalide and donepezil 27. Caffeine 28. Cannabidiol 29. Carvedilol 30. CDNF 31. Ceftriaxone 32. CEP-1347 33. cgpMax 34. Chlorogenic acid 35. Cinpanemab 36. Citalopram 37. CNM-Au8 38. CoQ10 39. CoQ10 and creatine 40. CoQ10 and vitamin E 41. Creatine 42. Creatine and minocycline 43. CST-2032 44. Cu(II)ATSM 45. D-Serine 46. DA-9805 47. Deferasirox 48. Deferiprone 49. Deprenyl and levodopa 50. Deprenyl and tocopherol 51. Dimethylfumarate 52. Diroximel fumarate 53. DL-3-n-butylphtalide 54. DNL-151 55. Donepezil 56. Duloxetine 57. Epothilone D 58. ESB1609 59. Exenatide 60. Exifone 61. Faecal microbiota transfer 62. Faecal microbiota transplant 63. Fasudil 64. FB-101/1ST-102 65. Febuxostat 66. Febuxostat and inosine 67. Felodipine 68. Fenofibrate 69. Fingolimod 70. Glibenclamide 71. Glutathione 72. GM1 Ganglioside 73. GPI-1485 74. Green tea polyphenol 75. GRF6021 76. Herbal chinese medicine (Roucongrong and adjuvant) 77. Herbal medicinal mixture (Roucongrong, Heshouwu, etc.) 78. Homotaurine (tramiprosate) 79. Hydrogen gas inhalation 80. Hydrogen water 81. Hypoestoxide 82. Ibuprofen 83. Idebenone 84. IkT-148009 85. Inosine 86. Intranasal insulin 87. Inzomelid 88. Isradipine 89. Isradipine CR 90. Istradefylline 91. ITI-214 92. K0706/SCC-138 93. Ketone ester 94. KM819 95. L-dopa with carbidopa 96. Lazabemide 97. Levetiracetam 98. Licorice 99. Lingzhi (Ganoderma) 100. Liquigen MCT 101. Liraglutide | 1. Lithium 2. Lixisenatide 3. Lonafarnib 4. Lovastatin 5. Lu AF82422 6. Mannitol 7. MEDI1341 8. Medopa with pramipexole 9. Melatonin 10. Memantine 11. Metabolic cofactor supplementation (serine, L-carnitine tartrate, N-acetylcysteine and nicotinamide riboside) 12. Metformin 13. Minocycline 14. MitoQ 15. Montelukast 16. N-3 PUFA (e.g. Neuroaspis PLP10™) 17. N-Acetyl cysteine 18. NBTX-001 19. NE3107 20. Neflamapimod 21. Niacin (vitamin B3) 22. Nicotinamide riboside 23. Nicotine 24. Nilotinib 25. Nilvadipine 26. NLY-01 27. NNI-362 28. NNI-370 29. Nortriptyline 30. NPT-200-11/UCB-0599 31. NPT088 32. NPT520-34 33. Olesoxime 34. Omega-3 fatty acids and vitamin E 35. Oxaloacetate 36. PDGF-BB/sNN0031 37. Pergolide 38. Phenylbutyrate 39. Pioglitazone 40. Plerixafor 41. PMX205 42. Pomalidomide 43. Pramipexole 44. Prasinezumab 45. PRIM-DJ2727 46. Probiotic compound 47. Probiotic supplements 48. Propionic acid 49. PT320 50. Quetiapine 51. Radotinib HCl 52. Rasagiline 53. Recipe for nourishing Gan-Shen 54. Resveratrol 55. Rifabutin 56. Rifaximin 57. Riluzole 58. Rivastigmine 59. Ropinirole 60. RP-323 61. Rucaparib 62. Salbutamol/albuterol 63. Saracatinib 64. Sargramostim 65. Selegiline 66. Semaglutide 67. Senicapoc 68. Simvastatin 69. Sulforaphane 70. Tacrolimus 71. Talineuren 72. TCH346 73. Telmisartan 74. Terazosin 75. Thalidomide 76. Theracurmin 77. Thymoquinone 78. Tocovid Suprabio (HOV-12020) 79. Trehalose 80. Trigonella foenum-graecum L seeds 81. UB-312 82. UCB0599 83. UCB7853 84. UDCA 85. Venglustat 86. Vinpocetine 87. Vitamin B12/methylcobalamin 88. Vitamin D 89. Vitamin K2 90. VIUSID/ALZER (Nutritional supplement) 91. Whey protein 92. WIN-1001X (Herbal mixture) 93. Xifeng Dingchan Pill 94. Young blood plasma 95. YTX-7739 96. Zolpidem 97. Zonisamide |

Abbreviations: CoQ10: Coenzyme Q10; MCT: Medium chain triglycerides; PUFA: Polyunsaturated fatty acids, UDCA: Ursodeoxycholic acid.

Supplementary Figure 2. Flowchart of the treatment selection process.

- 3 from APM trial
- 47 from iLCT candidates
- 3 from PubMed searches
- 81 from Hope List
- 60 from UoP scoping review
- 4 invited suggestions
- 10 highest scores
- 4 additional compounds to ensure ≥ 2 compounds/subgroup

**Selected compounds**

**3: Telmisartan. Terazosin, and UDCA**

Abbreviations: APM: Australian Parkinson’s Mission trial; iLCT: international Linked Clinical Trials (initiative); UDCA: ursodeoxycholic acid; UoP: University of Plymouth.

Supplementary Table 4. EJS ACT-PD Consortium

| **Member** | **Role** | **Organization** |
| --- | --- | --- |
| Thomas Foltynie | Co-Lead | University College London, London, UK |
| Camille B Carroll | Co-Lead | Newcastle University, Newcastle, UK;  University of Plymouth, Plymouth, UK |
| Roger Barker | Chair | University of Cambridge, Cambridge, UK |
| James Carpenter | Chair | MRC Clinical Trials Unit at UCL, London, UK |
| Yoav Ben-Shlomo | Member | University of Bristol, Bristol, UK |
| Mark Edwards | Member | St George’s University of London, London, UK |
| Alan Whone | Member | University of Bristol, Bristol, UK |
| Carl Counsell | Member | University of Aberdeen, Aberdeen, UK |
| Caroline S Clarke | Member | University College London, London, UK |
| Matthew Burnell | Member | MRC Clinical Trials Unit at UCL, London, UK |
| Kate Hockey | PPI | Expert through Experience |
| Anna Jewell | PPI | Expert through Experience |
| Priti Gros | Member | University of Toronto, Toronto, Canada |
| Tom Barber | ECR | University of Oxford, Oxford, UK |
| Anette Schrag | Chair | University College London, London, UK |
| Rimona S Weil | Deputy Chair | University College London, London, UK |
| Caroline H Williams-Gray | Member | University of Cambridge, UK |
| Michele T Hu | Member | University of Oxford, Oxford, UK |
| Lynn Rochester | Member | Newcastle University, Newcastle, UK |
| Paola Piccini | Member | Imperial College London, London UK |
| Henrik Zetterberg | Member | University College London, London, UK / University of Gothenburg, Mölndal, Sweden |
| Alastair Noyce | Member | Queen Mary University of London, London UK |
| Michael Lawton | Member | University of Bristol, Bristol, UK |
| Ashwani Jha | Member | University College London, London, UK |
| Brook Huxford | Member | Queen Mary University of London, London, UK |
| Shlomi Haar Millo | Member | Imperial College London, London, UK |
| K. Ray Chaudhuri | Member | King's College London, London, UK |
| Carroll Siu | PPI | Expert through Experience |
| Michèle Bartlett | PPI | Expert through Experience |
| Kuhan Pushparatnam | PPI | Expert through Experience |
| Daniel van Wamelen | ECR | King's College London, London, UK |
| Anthony HV Schapira | Co-Chair | University College London, London, UK |
| Oliver Bandmann | Co-Chair | University of Sheffield, Sheffield, UK |
| Simon Stott | Member | Cure Parkinson's, London, UK |
| George Tofaris | Member | University of Oxford, Oxford, UK |
| Esther Sammler | Member | University of Dundee, Dundee, UK |
| Heather Mortiboys | Member | University of Sheffield, Sheffield, UK |
| Li Wei | Member | University College London, London, UK |
| Alan Wong | Member | Royal Free Hospital NHS Foundation Trust, London, UK |
| Susan Duty | Member | King's College London, London, UK |
| David Dexter | Member | Parkinson's UK, London, UK |
| Edwin Jabbari | ECR | University College London, London, UK |
| Stephen Mullin | Chair | University of Plymouth, Plymouth, UK |
| Huw Morris | Member | University College London, London, UK |
| David Breen | Member | University of Edinburgh, Edinburgh, UK |
| Christian Lambert | Member | University College London, London, UK |
| Prasad Korlipara | Member | University College London, London, UK |
| Monty Silverdale | Member | University of Manchester, Manchester, UK |
| Kailash Bhatia | Member | University College London, London, UK |
| Alison Yarnall | Member | Newcastle University, Newcastle, UK |
| Raj Khengar | Member | University College London, London, UK |
| Helen Collins | Member | Nat National Institute of Health Research Clinical Research Network, UK |
| Fleur Hudson | Member | MRC Clinical Trials Unit at UCL, London, UK. |
| Rebecca Croucher | Member | National Institute of Health Research Clinical Research Network, UK |
| Sandra Bartolomeu-Pires | Member | Southhampton NHS Foundation Trust, Southampton, UK |
| Veena Agarwal | Member | Southhampton NHS Foundation Trust, Southampton, UK |
| Jennifer Allison | Member | National Institute of Health Research Clinical Research Network, UK |
| Jodie Forbes | PPI | Expert through Experience |
| Alex Edwards | Member | Parkinson’s UK, London, UK |
| Sheila Wonnacott | PPI | Expert through Experience |
| Dilan Athauda | ECR | University College London, London, UK |
| Joy Duffen | Co-Chair | Cure Parkinson’s, London, UK |
| Sonia Gandhi | Co-Chair | University College London, London, UK |
| Emily Henderson | Member | University of Bristol, Bristol, UK |
| Jen Black | Member | University College London, London, UK |
| Karen Matthews | Member | National Institute of Health Research Clinical Research Network, UK |
| Vince Greaves | Member | University College London, London, UK |
| Eric Deeson | PPI | Expert through Experience |
| Laurel Miller | PPI | Expert through Experience |
| Joel Handley | ECR | Salford Royal NHS Foundation Trust, UK |
| Helen Matthews | Member | Cure Parkinson’s, London, UK |
| Kevin McFarthing | Chair | Expert through Experience |
| Amit Batla | Member | University College London, London, UK |
| Nikul Bakshi | Member | Parkinson's UK, London, UK |
| Miriam Parry | Member | Kings College Hospital NHS Foundation Trust, London, UK |
| Natasha Ratcliffe | Member | Independent Advisor, Manchester, UK |
| Cheney Drew | Member | Cardiff University, Cardiff, UK |
| Naveena Kapur | Member | Parkinson's UK, London, UK |
| Anaya Navangul | Member | Cure Parkinson's, London, UK |
| Shafaq Ali | Member | Expert through Experience |
| Katherine Fletcher | Member | Parkinson's UK, London, UK |
| Claire Bale | Member | Parkinson's UK, London, UK |
| Cristina Gonzalez-Robles | Member | University College London, London, UK |
| Marie-Louise Zeissler | Member | University of Plymouth, Plymouth, UK |
| Georgia Mills | Member | University College London, London, UK |
| Romy Ellis-Doyle | Member | University College London, London, UK |
| Sally Collins | Member | University of Plymouth, Plymouth, UK |
| Rebecca Petty | Member | University of Plymouth, Plymouth, UK |

Abbreviations: ECR: Early career researcher; PPI: Patient and public involvement representative

Supplementary references (from Table 2)

1. Pang T, Wang J, Benicky J, Sánchez-Lemus E, Saavedra JM. Telmisartan directly ameliorates the neuronal inflammatory response to IL-1β partly through the JNK/c-Jun and NADPH oxidase pathways. J Neuroinflammation. 2012;9:1–19.

2. Rodriguez-Perez AI, Sucunza D, Pedrosa MA, Garrido-Gil P, Kulisevsky J, Lanciego JL, et al. Angiotensin Type 1 Receptor Antagonists Protect Against Alpha-Synuclein-Induced Neuroinflammation and Dopaminergic Neuron Death. Neurotherapeutics. 2018;15(4):1063–81.

3. Garrido-Gil P, Joglar B, Rodriguez-Perez AI, Guerra MJ, Labandeira-Garcia JL. Involvement of PPAR-γ in the neuroprotective and anti-inflammatory effects of angiotensin type 1 receptor inhibition: Effects of the receptor antagonist telmisartan and receptor deletion in a mouse MPTP model of Parkinson’s disease. J Neuroinflammation. 2012;9:1–16.

4. Ray B, Ramesh G, Verma SR, Ramamurthy S, Tuladhar S, Mahalakshmi AM, et al. Effects of Telmisartan, an AT1 receptor antagonist, on mitochondria-specific genes expression in a mouse MPTP model of Parkinsonism. Front Biosci - Landmark. 2021;26(8):262–71.

5. Sekar S, Mani S, Rajamani B, Manivasagam T, Thenmozhi AJ, Bhat A, et al. Telmisartan Ameliorates Astroglial and Dopaminergic Functions in a Mouse Model of Chronic Parkinsonism. Neurotox Res. 2018;34(3):597–612.

6. Tong Q, Wu L, Jiang T, Ou Z, Zhang Y, Zhu D. Inhibition of endoplasmic reticulum stress-activated IRE1α-TRAF2-caspase-12 apoptotic pathway is involved in the neuroprotective effects of telmisartan in the rotenone rat model of Parkinson’s disease. Eur J Pharmacol. 2016;776:106–15.

7. Garrido-Gil P, Valenzuela R, Villar-Cheda B, Lanciego JL, Labandeira-Garcia JL. Expression of angiotensinogen and receptors for angiotensin and prorenin in the monkey and human substantia nigra: An intracellular renin-angiotensin system in the nigra. Brain Struct Funct. 2013;218(2):373–88.

8. Kamath T, Abdulraouf A, Burris SJ, Langlieb J, Gazestani V, Nadaf NM, et al. Single-cell genomic profiling of human dopamine neurons identifies a population that selectively degenerates in Parkinson’s disease. Nat Neurosci [Internet]. 2022 May 5;25(5):588–95. Available from: https://www.nature.com/articles/s41593-022-01061-1

9. Chen X, Zhao C, Li X, Wang T, Li Y, Cao C, et al. Terazosin activates Pgk1 and Hsp90 to promote stress resistance. Nat Chem Biol [Internet]. 2015 Jan;11(1):19–25. Available from: http://www.ncbi.nlm.nih.gov/pubmed/25383758

10. Cai R, Welsh MJ, Liu L, Cai R, Zhang Y, Simmering JE, et al. Enhancing glycolysis attenuates Parkinson’s disease progression in models and clinical databases. J Clin Invest. 2019;129(10):4539–49.

11. Qi H, Shen D, Jiang C, Wang H, Chang M. Ursodeoxycholic acid protects dopaminergic neurons from oxidative stress via regulating mitochondrial function, autophagy, and apoptosis in MPTP/MPP+-induced Parkinson’s disease. Neurosci Lett [Internet]. 2021;741(November 2020):135493. Available from: https://doi.org/10.1016/j.neulet.2020.135493

12. Mortiboys H, Furmston R, Bronstad G, Aasly J, Elliott C, Bandmann O. UDCA exerts beneficial effect on mitochondrial dysfunction in LRRK2 G2019S carriers and in vivo. Neurology. 2015;85(10):846–52.

13. Abdelkader NF, Safar MM, Salem HA. Ursodeoxycholic Acid Ameliorates Apoptotic Cascade in the Rotenone Model of Parkinson’s Disease: Modulation of Mitochondrial Perturbations. Mol Neurobiol. 2016;53(2):810–7.

14. Lin HC, Tseng YF, Shen AL, Chao JCJ, Hsu CY, Lin HL. Association of Angiotensin Receptor Blockers with Incident Parkinson Disease in Patients with Hypertension: A Retrospective Cohort Study. Am J Med [Internet]. 2022;135(8):1001–7. Available from: https://doi.org/10.1016/j.amjmed.2022.04.029

15. Jo Y, Kim S, Ye BS, Lee E, Yu YM. Protective Effect of Renin-Angiotensin System Inhibitors on Parkinson’s Disease: A Nationwide Cohort Study. Front Pharmacol. 2022;13(March):1–11.

16. Labandeira CM, Pedrosa MA, Quijano A, Valenzuela R, Garrido-Gil P, Sanchez-Andrade M, et al. Angiotensin type-1 receptor and ACE2 autoantibodies in Parkinson´s disease. npj Park Dis. 2022;8(1).

17. Simmering JE, Welsh MJ, Liu L, Narayanan NS, Pottegård A. Association of Glycolysis-Enhancing α-1 Blockers with Risk of Developing Parkinson Disease. JAMA Neurol. 2021;78(4):407–13.

18. Sasane R, Bartels A, Field M, Sierra MI, Duvvuri S, Gray DL, et al. Parkinson disease among patients treated for benign prostatic hyperplasia with α1 adrenergic receptor antagonists. J Clin Invest. 2021;131(11).

19. Simmering JE, Welsh MJ, Schultz J, Narayanan NS. Use of Glycolysis-Enhancing Drugs and Risk of Parkinson’s Disease. Mov Disord. 2022;37(11):2210–6.

20. Li P, Killinger BA, Ensink E, Beddows I, Yilmaz A, Lubben N, et al. Gut microbiota dysbiosis is associated with elevated bile acids in parkinson’s disease. Metabolites. 2021;11(1):1–15.

21. Shao Y, Li T, Liu Z, Wang X, Xu X, Li S, et al. Comprehensive metabolic profiling of Parkinson’s disease by liquid chromatography-mass spectrometry. Mol Neurodegener. 2021;16(1):1–15.

22. Mitsui T, Kuroda Y, Ph D, Adachi K, Kaji R. Effect of angiotensin II type 1-receptor blocker candesartan on hypertensive Parkinson’s disease. Candesartan Park Dis. 2009;1:1–5.

23. Schultz JL, Brinker AN, Xu J, Ernst SE, Tayyari F, Rauckhorst AJ, et al. A pilot to assess target engagement of terazosin in Parkinson’s disease. Park Relat Disord [Internet]. 2022;94(November 2021):79–83. Available from: https://doi.org/10.1016/j.parkreldis.2021.11.022

24. Arena G, Landoulsi Z, Grossmann D, Payne T, Vitali A, Delcambre S, et al. Polygenic Risk Scores Validated in Patient-Derived Cells Stratify for Mitochondrial Subtypes of Parkinson’s Disease. Ann Neurol. 2024;96(1):133–49.
