# Supplementary material for: Treatment Selection and Prioritization for the EJS ACT‐PD MAMS Trial Platform
Source: Mov Disord. 2025 Apr 18;40(7):1307–17. doi: 10.1002/mds.30190 (PMC12273612; doi:10.1002/mds.30190)
Supplement: Supplementary file 1 — Data S1 Supporting Information. [file MDS-40-1307-s001.zip › mds30190-sup-0005-Supplementary references.docx]

Supplementary references (from Table 2)

1. Pang T, Wang J, Benicky J, Sánchez-Lemus E, Saavedra JM. Telmisartan directly ameliorates the neuronal inflammatory response to IL-1β partly through the JNK/c-Jun and NADPH oxidase pathways. J Neuroinflammation. 2012;9:1–19.

2. Cai R, Welsh MJ, Liu L, Cai R, Zhang Y, Simmering JE, et al. Enhancing glycolysis attenuates Parkinson’s disease progression in models and clinical databases. J Clin Invest. 2019;129(10):4539–49.

3. Chun HS, Low WC. Ursodeoxycholic acid suppresses mitochondria-dependent programmed cell death induced by sodium nitroprusside in SH-SY5Y cells. Toxicology [Internet]. 2012;292(2–3):105–12. Available from: http://dx.doi.org/10.1016/j.tox.2011.11.020

4. Mortiboys H, Aasly J, Bandmann O. Ursocholanic acid rescues mitochondrial function in common forms of familial Parkinson’s disease. Brain. 2013;136(10):3038–50.

5. Qi H, Shen D, Jiang C, Wang H, Chang M. Ursodeoxycholic acid protects dopaminergic neurons from oxidative stress via regulating mitochondrial function, autophagy, and apoptosis in MPTP/MPP+-induced Parkinson’s disease. Neurosci Lett [Internet]. 2021;741(November 2020):135493. Available from: https://doi.org/10.1016/j.neulet.2020.135493

6. Garrido-Gil P, Joglar B, Rodriguez-Perez AI, Guerra MJ, Labandeira-Garcia JL. Involvement of PPAR-γ in the neuroprotective and anti-inflammatory effects of angiotensin type 1 receptor inhibition: Effects of the receptor antagonist telmisartan and receptor deletion in a mouse MPTP model of Parkinson’s disease. J Neuroinflammation. 2012;9:1–16.

7. Sekar S, Mani S, Rajamani B, Manivasagam T, Thenmozhi AJ, Bhat A, et al. Telmisartan Ameliorates Astroglial and Dopaminergic Functions in a Mouse Model of Chronic Parkinsonism. Neurotox Res. 2018;34(3):597–612.

8. Sathiya S, Ranju V, Kalaivani P, Priya RJ, Sumathy H, Sunil AG, et al. Telmisartan attenuates MPTP induced dopaminergic degeneration and motor dysfunction through regulation of a-synuclein and neurotrophic factors (BDNF and GDNF) expression in C57BL/6J mice. Neuropharmacology [Internet]. 2013;73:98–110. Available from: http://dx.doi.org/10.1016/j.neuropharm.2013.05.025

9. Ray B, Ramesh G, Verma SR, Ramamurthy S, Tuladhar S, Mahalakshmi AM, et al. Effects of Telmisartan, an AT1 receptor antagonist, on mitochondria-specific genes expression in a mouse MPTP model of Parkinsonism. Front Biosci - Landmark. 2021;26(8):262–71.

10. Rodriguez-Perez AI, Sucunza D, Pedrosa MA, Garrido-Gil P, Kulisevsky J, Lanciego JL, et al. Angiotensin Type 1 Receptor Antagonists Protect Against Alpha-Synuclein-Induced Neuroinflammation and Dopaminergic Neuron Death. Neurotherapeutics. 2018;15(4):1063–81.

11. Tong Q, Wu L, Jiang T, Ou Z, Zhang Y, Zhu D. Inhibition of endoplasmic reticulum stress-activated IRE1α-TRAF2-caspase-12 apoptotic pathway is involved in the neuroprotective effects of telmisartan in the rotenone rat model of Parkinson’s disease. Eur J Pharmacol. 2016;776:106–15.

12. Mortiboys H, Furmston R, Bronstad G, Aasly J, Elliott C, Bandmann O. UDCA exerts beneficial effect on mitochondrial dysfunction in LRRK2 G2019S carriers and in vivo. Neurology. 2015;85(10):846–52.

13. Abdelkader NF, Safar MM, Salem HA. Ursodeoxycholic Acid Ameliorates Apoptotic Cascade in the Rotenone Model of Parkinson’s Disease: Modulation of Mitochondrial Perturbations. Mol Neurobiol. 2016;53(2):810–7.

14. Garrido-Gil P, Valenzuela R, Villar-Cheda B, Lanciego JL, Labandeira-Garcia JL. Expression of angiotensinogen and receptors for angiotensin and prorenin in the monkey and human substantia nigra: An intracellular renin-angiotensin system in the nigra. Brain Struct Funct. 2013;218(2):373–88.

15. Zawada WM, Mrak RE, Biedermann JA, Palmer QD, Gentleman SM, Aboud O, et al. Loss of angiotensin II receptor expression in dopamine neurons in Parkinson’s disease correlates with pathological progression and is accompanied by increases in Nox4- and 8-OH guanosine-related nucleic acid oxidation and caspase-3 activation. Acta Neuropathol Commun. 2015;3:9.

16. Kamath T, Abdulraouf A, Burris SJ, Langlieb J, Gazestani V, Nadaf NM, et al. Single-cell genomic profiling of human dopamine neurons identifies a population that selectively degenerates in Parkinson’s disease. Nat Neurosci [Internet]. 2022 May 5;25(5):588–95. Available from: https://www.nature.com/articles/s41593-022-01061-1

17. Carling PJ, Mortiboys H, Green C, Mihaylov S, Sandor C, Schwartzentruber A, et al. Deep phenotyping of peripheral tissue facilitates mechanistic disease stratification in sporadic Parkinson’s disease. Prog Neurobiol [Internet]. 2020;187(June 2019):101772. Available from: https://doi.org/10.1016/j.pneurobio.2020.101772

18. Michel MC, Foster C, Brunner HR, Liu L. A systematic comparison of the properties of clinically used angiotensin II type 1 receptor antagonists. Pharmacol Rev [Internet]. 2013;65(2):809–48. Available from: https://doi.org/10.1124/pr.112.007278

19. Shao J, Nangaku M, Inagi R, Kato H, Noiri E, Fujita T, et al. Receptor—independent intracellular radical scavenging activity of angiotensin II receptor blocker. Japanese J Nephrol. 2006;48(3):228–33.

20. Kurtz TW, Pravenec M. Molecule-specific effects of angiotensin II-receptor blockers independent of the renin-angiotensin system. Am J Hypertens. 2008;21(8):852–9.

21. Stangier J, Schmid J, Türck D, Switek H, Verhagen A, Peeters PA, et al. Absorption, metabolism, and excretion of intravenously and orally administered [14C]telmisartan in healthy volunteers. J Clin Pharmacol [Internet]. 2000 Dec;40(12 Pt 1):1312–22. Available from: http://www.ncbi.nlm.nih.gov/pubmed/11185629

22. Noda A, Fushiki H, Murakami Y, Sasaki H, Miyoshi S, Kakuta H, et al. Brain penetration of telmisartan, a unique centrally acting angiotensin II type 1 receptor blocker, studied by PET in conscious rhesus macaques. Nucl Med Biol [Internet]. 2012;39(8):1232–5. Available from: http://dx.doi.org/10.1016/j.nucmedbio.2012.06.012

23. Ho JK, Moriarty F, Manly JJ, Larson EB, Evans DA, Rajan KB, et al. Blood-Brain Barrier Crossing Renin-Angiotensin Drugs and Cognition in the Elderly: A Meta-Analysis. Hypertension. 2021;78(3):629–43.

24. Glodzik L, Santisteban MM. Blood-Brain Barrier Crossing Renin-Angiotensin System Drugs: Considerations for Dementia and Cognitive Decline. Hypertension. 2021;78(3):644–6.

25. Villapol S, Janatpour ZC, Affram KO, Symes AJ. The Renin Angiotensin System as a Therapeutic Target in Traumatic Brain Injury. Neurotherapeutics [Internet]. 2023;20(6):1565–91. Available from: https://doi.org/10.1007/s13311-023-01435-8

26. Schultz JL, Brinker AN, Xu J, Ernst SE, Tayyari F, Rauckhorst AJ, et al. A pilot to assess target engagement of terazosin in Parkinson’s disease. Park Relat Disord [Internet]. 2022;94(November 2021):79–83. Available from: https://doi.org/10.1016/j.parkreldis.2021.11.022

27. Schultz JL, Gander PE, Workman CD, Ponto LL, Cross S, Nance CS, et al. A pilot dose-finding study of Terazosin in humans. medRxiv Prepr Serv Heal Sci [Internet]. 2024 May 22; Available from: http://www.ncbi.nlm.nih.gov/pubmed/38826433

28. Sathe AG, Tuite P, Chen C, Ma Y, Chen W, Cloyd J, et al. Pharmacokinetics, Safety, and Tolerability of Orally Administered Ursodeoxycholic Acid in Patients With Parkinson’s Disease—A Pilot Study. J Clin Pharmacol. 2020;60(6):744–50.

29. Parry GJ, Rodrigues CMP, Aranha MM, Hilbert SJ, Davey C, Kelkar P, et al. Safety, tolerability, and cerebrospinal fluid penetration of ursodeoxycholic acid in patients with amyotrophic lateral sclerosis. Clin Neuropharmacol. 2010;33(1):17–21.

30. Payne T, Appleby M, Buckley E, van Gelder LMA, Mullish BH, Sassani M, et al. A Double-Blind, Randomized, Placebo-Controlled Trial of Ursodeoxycholic Acid (UDCA) in Parkinson’s Disease. Mov Disord. 2023;38(8):1493–502.

31. Lin HC, Tseng YF, Shen AL, Chao JCJ, Hsu CY, Lin HL. Association of Angiotensin Receptor Blockers with Incident Parkinson Disease in Patients with Hypertension: A Retrospective Cohort Study. Am J Med [Internet]. 2022;135(8):1001–7. Available from: https://doi.org/10.1016/j.amjmed.2022.04.029

32. Jo Y, Kim S, Ye BS, Lee E, Yu YM. Protective Effect of Renin-Angiotensin System Inhibitors on Parkinson’s Disease: A Nationwide Cohort Study. Front Pharmacol. 2022;13(March):1–11.

33. Labandeira CM, Pedrosa MA, Quijano A, Valenzuela R, Garrido-Gil P, Sanchez-Andrade M, et al. Angiotensin type-1 receptor and ACE2 autoantibodies in Parkinson´s disease. npj Park Dis. 2022;8(1).

34. Simmering JE, Welsh MJ, Liu L, Narayanan NS, Pottegård A. Association of Glycolysis-Enhancing α-1 Blockers with Risk of Developing Parkinson Disease. JAMA Neurol. 2021;78(4):407–13.

35. Sasane R, Bartels A, Field M, Sierra MI, Duvvuri S, Gray DL, et al. Parkinson disease among patients treated for benign prostatic hyperplasia with α1 adrenergic receptor antagonists. J Clin Invest. 2021;131(11).

36. Simmering JE, Welsh MJ, Schultz J, Narayanan NS. Use of Glycolysis-Enhancing Drugs and Risk of Parkinson’s Disease. Mov Disord. 2022;37(11):2210–6.

37. Li P, Killinger BA, Ensink E, Beddows I, Yilmaz A, Lubben N, et al. Gut microbiota dysbiosis is associated with elevated bile acids in parkinson’s disease. Metabolites. 2021;11(1):1–15.

38. Shao Y, Li T, Liu Z, Wang X, Xu X, Li S, et al. Comprehensive metabolic profiling of Parkinson’s disease by liquid chromatography-mass spectrometry. Mol Neurodegener. 2021;16(1):1–15.

39. Mitsui T, Kuroda Y, Ph D, Adachi K, Kaji R. Effect of angiotensin II type 1-receptor blocker candesartan on hypertensive Parkinson’s disease. Candesartan Park Dis. 2009;1:1–5.

40. Arena G, Landoulsi Z, Grossmann D, Payne T, Vitali A, Delcambre S, et al. Polygenic Risk Scores Validated in Patient-Derived Cells Stratify for Mitochondrial Subtypes of Parkinson’s Disease. Ann Neurol. 2024;96(1):133–49.
