# Supplementary material for: Treatment Selection and Prioritization for the EJS ACT‐PD MAMS Trial Platform
Source: Mov Disord. 2025 Apr 18;40(7):1307–17. doi: 10.1002/mds.30190 (PMC12273612; doi:10.1002/mds.30190)
Supplement: Supplementary file 1 — Data S1 Supporting Information. [file MDS-40-1307-s001.zip › mds30190-sup-0002-Supp Figure 1 color - for online version of article.pdf]

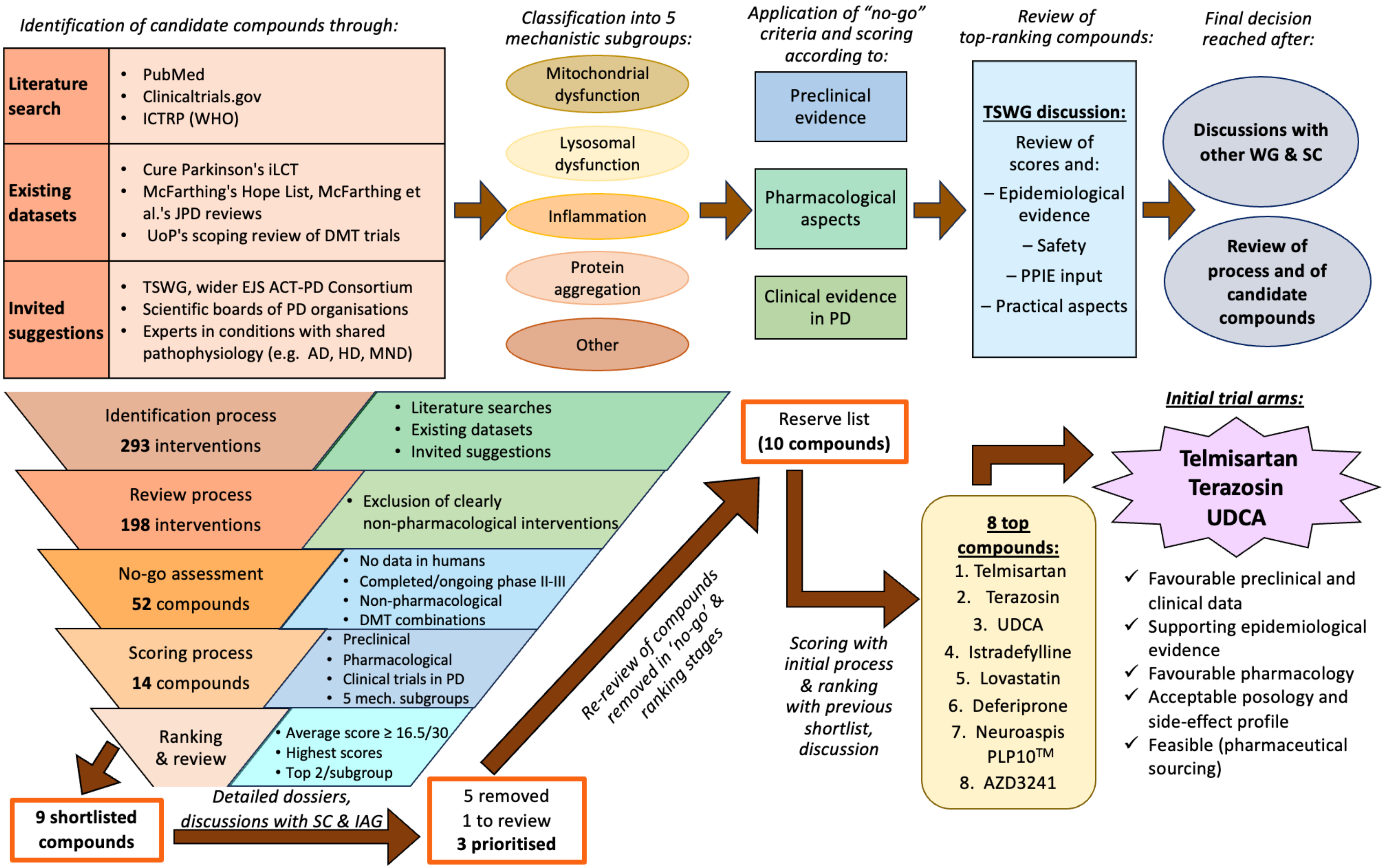
Identification of candidate compounds through:
Literature search
PubMed
Clinicaltrials.gov
ICTRP (WHO)
Existing datasets
Cure Parkinson's iLCT
McFarthing's Hope List, McFarthing et al.'s JPD reviews
UoP's scoping review of DMT trials
Invited suggestions
TSWG, wider EJS ACT-PD Consortium
Scientific boards of PD organisations
Experts in conditions with shared pathophysiology (e.g. AD, HD, MND)
Classification into 5 mechanistic subgroups:
Mitochondrial dysfunction
Lysosomal dysfunction
Inflammation
Protein aggregation
Other
Application of "no-go" criteria and scoring according to:
Preclinical evidence
Pharmacological aspects
Clinical evidence in PD
Review of top-ranking compounds:
TSWG discussion:
Review of scores and:
– Epidemiological evidence
– Safety
– PPIE input
– Practical aspects
Final decision reached after:
Discussions with other WG & SC
Review of process and of candidate compounds
Identification process
293 interventions
Literature searches
Existing datasets
Invited suggestions
Review process
198 interventions
Exclusion of clearly non-pharmacological interventions
No-go assessment
52 compounds
No data in humans
Completed/ongoing phase II-III
Non-pharmacological
DMT combinations
Scoring process
14 compounds
Preclinical
Pharmacological
Clinical trials in PD
5 mech. subgroups
Ranking & review
Average score ≥ 16.5/30
Highest scores
Top 2/subgroup
9 shortlisted compounds
Detailed dossiers, discussions with SC & IAG
5 removed
1 to review
3 prioritised
Re-review of compounds removed in 'no-go' & ranking stages
Reserve list (10 compounds)
Scoring with initial process & ranking with previous shortlist, discussion
8 top compounds:
1. Telmisartan
2. Terazosin
3. UDCA
4. Istradefylline
5. Lovastatin
6. Deferiprone
7. Neuroaspis PLP10™
8. AZD3241
Initial trial arms:
Telmisartan
Terazosin
UDCA
✓ Favourable preclinical and clinical data
✓ Supporting epidemiological evidence
✓ Favourable pharmacology
✓ Acceptable posology and side-effect profile
✓ Feasible (pharmaceutical sourcing)
