# Supplementary figures and images for: Treatment Selection and Prioritization for the EJS ACT‐PD MAMS Trial Platform
Source: Mov Disord. 2025 Apr 18;40(7):1307–17. doi: 10.1002/mds.30190 (PMC12273612; doi:10.1002/mds.30190)

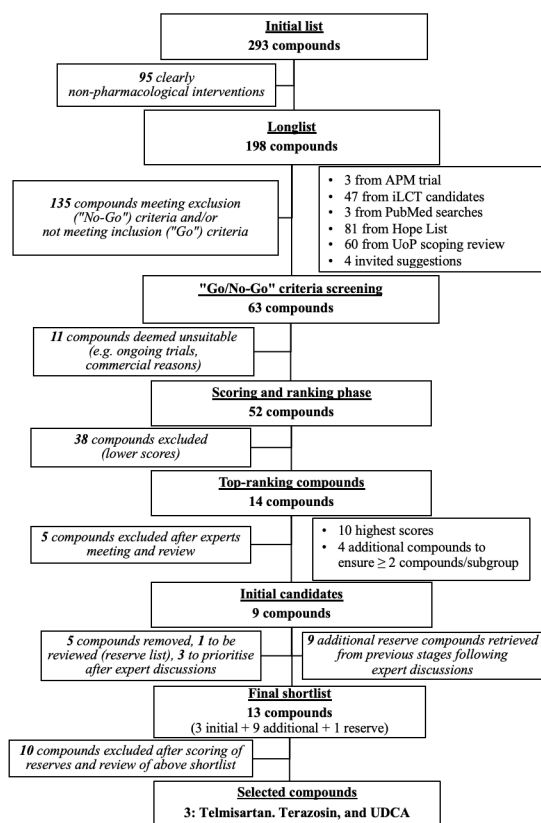

Supplement: Supplementary file 1 — Data S1 Supporting Information. [file MDS-40-1307-s001.zip › mds30190-sup-0003-Supplementary Figure 2 EJS ACT-PD treatment selection 2025.02.04.pdf]
